# NDFIP allows NEDD4/NEDD4L-induced AQP2 ubiquitination and degradation

**DOI:** 10.1371/journal.pone.0183774

**Published:** 2017-09-20

**Authors:** Christiane Trimpert, Daniel Wesche, Theun de Groot, Martha M. Pimentel Rodriguez, Victoria Wong, Dennis T. M. van den Berg, Lydie Cheval, Carolina A. Ariza, Alain Doucet, Igor Stagljar, Peter M. T. Deen

**Affiliations:** 1 Department of Physiology, Radboud University Medical Center, Nijmegen, The Netherlands; 2 Donnelly Centre for Cellular and Biomolecular Research, Departments of Biochemistry and Molecular Genetics, University of Toronto, Toronto, ON, Canada; 3 Sorbonne Universités, UPMC Univ Paris 06, INSERM, Université Paris Descartes, Sorbonne Paris Cité, CNRS, Centre de Recherche des Cordeliers, Paris, France; University of Pittsburgh, UNITED STATES

## Abstract

Regulation of our water homeostasis is fine-tuned by dynamic translocation of Aquaporin-2 (AQP2)-bearing vesicles to and from the plasma membrane of renal principal cells. Whereas binding of vasopressin to its type-2 receptor initiates a cAMP-protein kinase A cascade and AQP2 translocation to the apical membrane, this is counteracted by protein kinase C-activating hormones, resulting in ubiquitination-dependent internalization of AQP2. The proteins targeting AQP2 for ubiquitin-mediated degradation are unknown. In collecting duct mpkCCD cells, siRNA knockdown of NEDD4 and NEDD4L E3 ligases yielded increased AQP2 abundance, but they did not bind AQP2. Membrane Yeast Two-Hybrid assays using full-length AQP2 as bait, identified NEDD4 family interacting protein 2 (NDFIP2) to bind AQP2. NDFIP2 and its homologue NDFIP1 have PY motifs by which they bind NEDD4 family members and bring them close to target proteins. In HEK293 cells, NDFIP1 and NDFIP2 bound AQP2 and were essential for NEDD4/NEDD4L-mediated ubiquitination and degradation of AQP2, an effect not observed with PY-lacking NDFIP1/2 proteins. In mpkCCD cells, downregulation of NDFIP1, NEDD4 and NEDD4L, but not NDFIP2, increased AQP2 abundance. In mouse kidney, Ndfip1 and Ndfip2 mRNA distribution was similar and high in proximal tubules and collecting ducts, which was also found for NDFIP1 proteins. Our results reveal that NEDD4/NEDD4L mediate ubiquitination and degradation of AQP2, but that NDFIP proteins are needed to connect NEDD4/NEDD4L to AQP2. As NDFIP1/2 bind many NEDD4 family E3 ligases, which are implicated in several cellular processes, NDFIP1/2 may be the missing link for AQP2 ubiquitination and degradation from different subcellular locations.

## Introduction

Hypernatremia or hypovolemia lead to an increased releases of vasopressin (AVP) from the pituitary. Released AVP binds to and activates its type-2 receptor (AVPR2) in the basolateral membrane of collecting duct principal cells and triggers a cyclic AMP (cAMP) signalling cascade leading to a changed phosphorylation of Aquaporin-2 (AQP2) water channels. Consequently, AQP2-containing intracellular vesicles are redistributed from the cytosol to the apical membrane. Driven by an osmotic gradient, water then enters the cells through AQP2 and exits the cell via AQP3 and AQP4, which corrects blood tonicity and volume and results in concentrated urine [1].

These corrected osmo and volume balances normalize blood AVP levels, which subsequently induces the internalization of AQP2 to storage vesicles and its lysosomal degradation, coinciding with a reduced water reabsorption. The fact that excessive renal water reabsorption and hyponatremia in SIADH, congestive heart failure, liver cirrhosis and preeclampsia coincide with elevated plasma membrane abundance of AQP2, whereas dehydration and hypernatremia in congenital and acquired forms of nephrogenic diabetes insipidus are due to insufficient plasma membrane abundance of AQP2 underscore the importance of a proper regulation of plasma membrane abundance of AQP2 [2].

In contrast to the well-studied regulatory system involved in AQP2 phosphorylation [3], very little is known about the players in AQP2 internalization. Earlier, we found that, following activation of protein kinase C (PKC), AQP2 was ubiquitinated and internalized [4]. However, ubiquitin ligases directly involved in this process are unknown. Ubiquitination is a posttranslational modification in which ubiquitin, a protein of 76 amino acids, is covalently coupled to a lysine of cellular protein, a process catalysed by an E3-ubiquitin protein ligase [5]. Lee et al. discovered a change in abundance of the BRE1B, CUL5 and NEDD4 in dDAVP-stimulated rat kidneys, and suggested a role for these E3 ligases in the regulation of water homeostasis [6]. However, AQP2 binding and functional evidence of involvement of any of these E3 ligases in AQP2 ubiquitination and degradation has not been reported.

Here, we provide evidence that NEDD4 and NEDD4L, also known as Nedd4-1 and Nedd4-2 respectively, can ubiquitinate AQP2, but not through direct binding of AQP2. Instead, using a Membrane Yeast Two-Hybrid (MYTH) assay to identify proteins interacting with full-length AQP2, we found that NEDD4 family interacting protein (NDFIP) 1 and 2 specifically interact with AQP2, are expressed in renal collecting ducts, and are essential for ubiquitination and degradation of AQP2 by NEDD4 and NEDD4L.

## Results

### NEDD4 and NEDD4L downregulate AQP2 expression, but not through direct interaction

**Fig 1:**
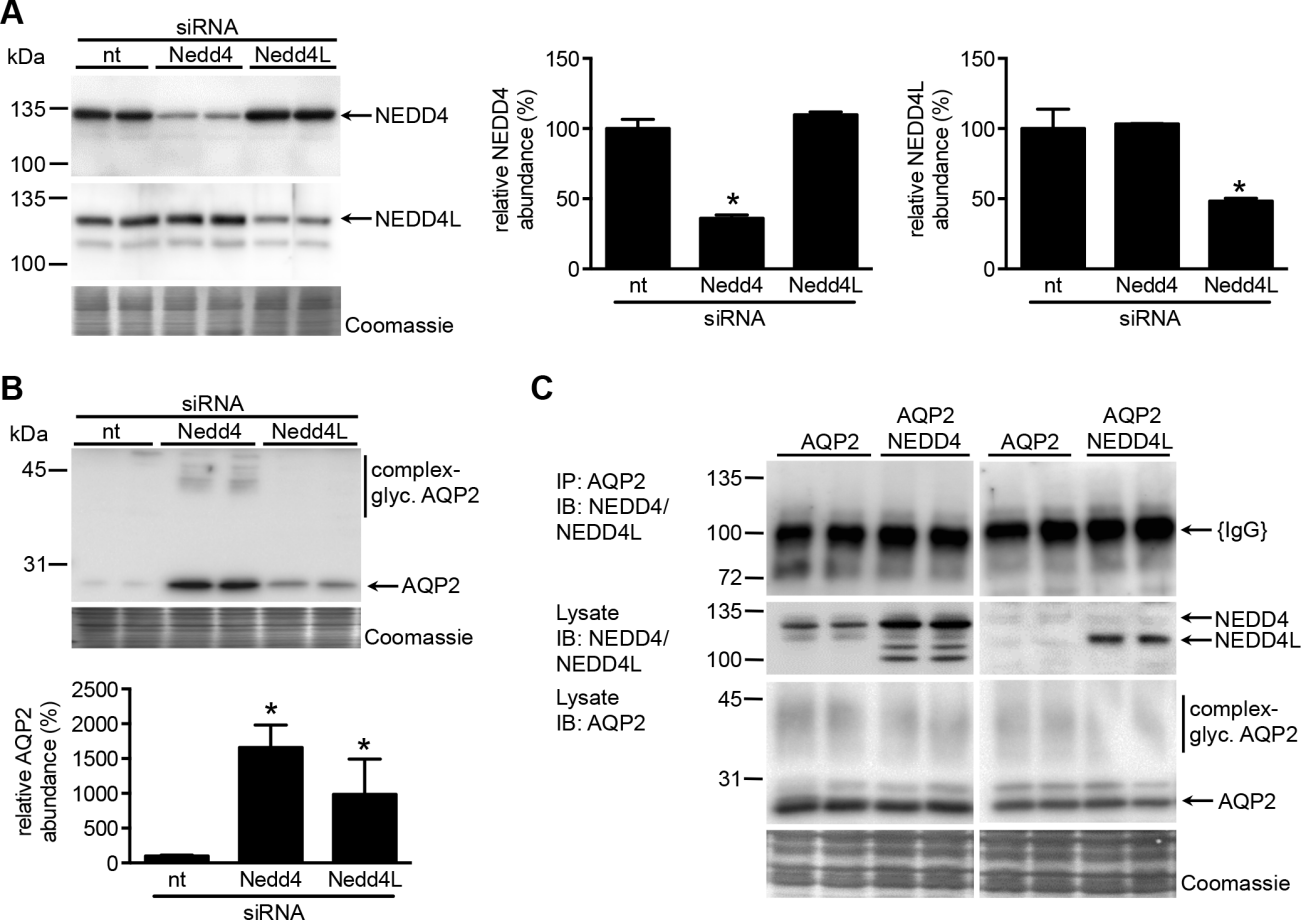
NEDD4 and NEDD4L downregulation increases AQP2 abundance. (A,B) *NEDD4/4L downregulation increases AQP2 abundance*. MpkCCD cells were transfected with non-targeting siRNAs (nt) or siRNAs against NEDD4 or NEDD4L, grown on filter for 4 days, treated with dDAVP for the last 72 hours, lysed, and subjected to immunoblotting for (A) NEDD4 and NEDD4L or (B) AQP2. (B) Semi-quantification of the signals (n = 6 from 3 independent experiments with duplicate samples) revealed significant and specific downregulation of NEDD4 and NEDD4L, leading to significantly-increased AQP2 abundances for NEDD4 and NEDD4L (*p<0.05 vs. nt-siRNA treatment). (C) *Lack of evidence that NEDD4/4L directly interact with AQP2*. HEK cells were transiently-transfected with expression constructs encoding AQP2 with or without constructs coding for NEDD4 or NEDD4L, grown for 2 days, lysed, and subjected to AQP2-immunoprecipitation (IP: α-AQP2). The IP-fractions and total lysate (indicated) were then immunoblotted for NEDD4 or NEDD4L (IB: α-NEDD4/NEDD4L) or AQP2 (IB: α-AQP2). Despite clear AQP2 and NEDD4/NEDD4L expression (lysates), NEDD4 nor NEDD4L was detected in the immunoprecipitates. Immunoglobulins used for immunoprecipitation ({IgG}) are indicated. Coomassie staining of the blots confirmed loading of protein equivalents. Molecular masses of marker proteins are indicated on the left (in kDa).

In the rat kidney inner medulla, withdrawal of dDAVP increased the abundance of the NEDD4 E3-ligase, while lithium-induced nephrogenic diabetes insipidus coincides with reduced NEDD4 abundance [6]. Furthermore, NEDD4 and NEDD4L regulate the plasma membrane abundance of the epithelial sodium channel ENaC in principal cells [7, 8], which also express AQP2. Mouse cortical collecting duct (mpkCCD) cells mimic renal principal cells, as they endogenously express and translocate AQP2 to the apical membrane in response to dDAVP[9]. Therefore, to test the potential role of these E3 ligases in AQP2 ubiquitination, internalization and degradation regulation, we transfected Nedd4/Nedd4L siRNAs into dDAVP-stimulated mpkCCD cells to test their effects on AQP2 abundance. Immunoblotting lysates for NEDD4 or NEDD4L expression showed the specificity of the siRNAs used, as NEDD4 abundance was significantly reduced with Nedd4, but not Nedd4L or non-targeting siRNAs and vice versa (Fig 1A). Subsequent immunoblotting for AQP2 revealed significantly-increased AQP2 abundances in cells transfected with Nedd4 or Nedd4L siRNAs as compared to cells transfected with non-targeting siRNA (Fig 1B). These data demonstrate a role for NEDD4 and NEDD4L in regulating AQP2 abundance in mpkCCD cells, possibly by affecting its ubiquitination-dependent degradation.

NEDD4 E3 ligases contain WW-domains, which bind to PY motifs in the PPxY amino acid stretch of target proteins [10], but AQP2 does not contain a PY motif. However, NEDD4 WW domains have also been reported to interact with phosphorylated S/T residues followed by a proline residue [11] and AQP2 is phosphorylated at the P262-flanking S261 upon internalization. Therefore, NEDD4/4L could downregulate AQP2 abundance by direct interaction with the water channel. As NEDD4 and NEDD4L expression in mpkCCD cells is rather low, AQP2 and NEDD4 or NEDD4L were co-expressed in HEK293 cells and subjected to an AQP2 co-immunoprecipitation assay. Immunoblotting for NEDD4 or NEDD4L on the AQP2 precipitate did not reveal any band, despite abundant expression of NEDD4/NEDD4L in the HEK293 lysates (Fig 1C). Altogether, we did not find any evidence that there is a direct interaction between NEDD4/NEDD4L and AQP2.

### NDFIP1 and NDFIP2 interact with AQP2

**Fig 2:**
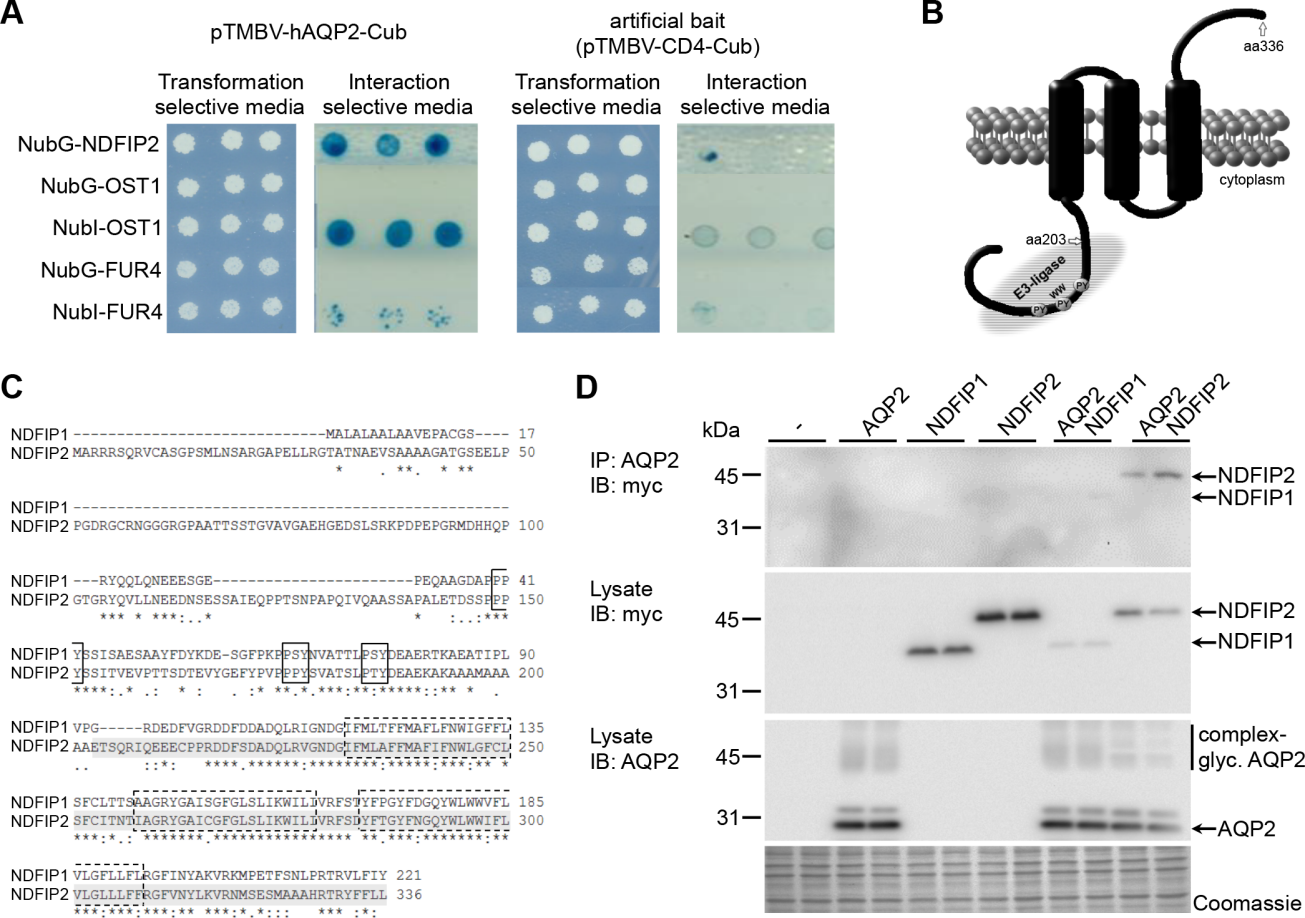
NDFIP2, a NEDD4 family interacting protein, binds to AQP2 in a MYTH assay. (A) *Specific interaction of NDFIP2 with AQP2*. Colony growth on interaction-selection media of yeast cells expressing NubG-NDFIP2 together with AQP2 indicates interaction (left two panels). No colony growth is detected with the artificial bait (CD4-Cub) a human integral membrane protein (right two panels). NubG-OST and NubI-OST serve as negative and positive controls for interaction in the ER-membrane, respectively, whereas NubG-FUR4 and NubI-FUR4 serve as negative and positive controls for interaction in the plasma membrane, respectively. These data confirm expression of AQP2 in the ER and plasma membrane of yeast cells and reveal the absence of self-activation. (B) *Topology and interaction elements of NDFIP2*. The part of NDFIP2 found to interact with AQP2 (amino acid 203–336, indicated with grey marking) covers part of the N-tail, the transmembrane domains (indicated with dashed line) and a part of the luminal/extracellular C-terminus. The PY elements which interact with the NEDD4/4L ww domains are indicated with a closed line. (C) *Alignment of the sequences of human NDFIP2 and NDFIP1*. Of the sequence of NDFIP2 (isoform 1; NP_061953.2) found to interact with AQP2 in the MYTH assay (aa 203–336, grey), 68% is identical (*) and 86% is similar (. or:) to that of NDFIP1 (NP_085048.1). The PPY-motifs (black rectangle) known to bind to NEDD4 and NEDD4L are not, but the transmembrane domains (dashed rectangle) are within the AQP2 binding region. (D) *NDFIP1 and NDFIP2 interact with AQP2*. HEK cells were transiently-transfected with an empty construct (-), or constructs encoding myc-tagged NDFIP1, NDFIP2 or AQP2 separately or combined (indicated on top), grown for 2 days, lysed and subjected to AQP2-immunoprecipitation (IP: α-AQP2). The IP-fractions (upper panel) and total lysates (indicated) were immunoblotted for NDFIP1 or -2 (IB: α-myc), or AQP2 (IB: α-AQP2). Only when co-expressed with AQP2, NDFIP1 and NDFIP2 were detected in the immunoprecipitate. Coomassie staining of the blots confirmed loading of protein equivalents. Molecular masses of proteins are indicated on the left (in kDa).

To potentially identify an E3 ligase or intermediate protein binding to AQP2, we used a Membrane Yeast Two-Hybrid (MYTH) assay to screen a human kidney cDNA library for interaction partners of full-length integral membrane AQP2. Following confirmation of interaction with re-transformation (Fig 2A), sequence analysis and an NCBI similarity BLAST search, we identified NEDD4 family interacting protein 2 (NDFIP2) as a binding partner of AQP2 (Fig 2A). NDFIP2 (NP_061953.2) is a predicted integral membrane protein of 336 amino acids with a cytosolic N-terminus and luminal/extracelllar C-terminus [12]. Its N-terminus contains three PY-motifs, which interact with WW-domains of NEDD4 and NEDD4L [13, 14]. The NDFIP2 clone from the MYTH screening spanned amino acids 203–336 (Fig 2B and 2C), indicating that the N-terminus and PY motifs of NDFIP2 are not involved in binding to AQP2. NDFIP2 could thus link NEDD4/NEDD4L to AQP2. Other proteins that gave positive results in our AQP2 MYTH, but were not confirmed, are given in S1 Table)

As the independent cDNAs of the library were ‘only’ screened one time for AQP2 interacting partners with our MYTH screening, related AQP2-interacting proteins were likely missing. Therefore, we performed a similarity BLAST search for NDFIP2. This revealed the existence of NDFIP1, which has a 68% amino acid identity and 86% similarity with NDFIP2 in the region binding AQP2 (Fig 2C). NDFIP1 also contains 3 PY-motifs and has been suggested to internalize membrane proteins, as it promoted the degradation of the divalent metal transporter (DMT1) membrane protein [15]. To analyse whether NDFIP1 and NDFIP2 interact with AQP2, myc-tagged NDFIP1 and NDFIP2 were co-expressed with AQP2 in HEK293. Myc-immunoblotting of AQP2-co-immunoprecipitated proteins revealed a clear band for NDFIP2 and a much weaker signal for NDFIP1, which correlates with the reduced expression of NDIP1 in the HEK293 lysates (Fig 2D).

### NDFIP1 and NDFIP2 confer NEDD4/NEDD4L-mediated AQP2 ubiquitination and degradation

**Fig 3:**
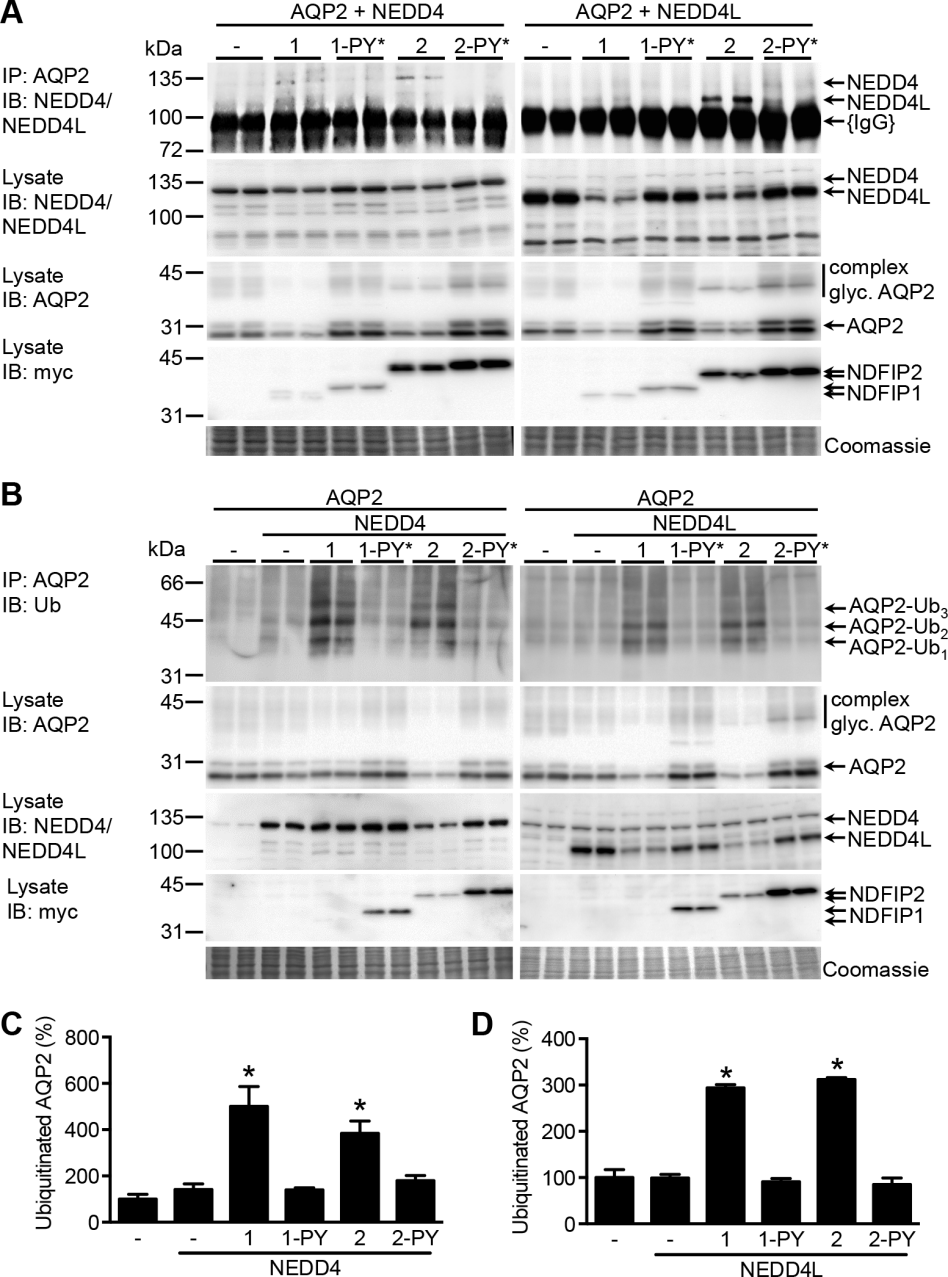
Linking of NEDD4 or NEDD4L to AQP2 by NDFIP1 or NDFIP2 mediates AQP2 ubiquitination. (A) *NDFIP1/2 link binding of NEDD4/NEDD4L to AQP2*. HEK293 cells transiently-expressing AQP2 and NEDD4 or AQP2 and NEDD4L with or without wildtype-NDFIP1/NDFIP2 (1, 2) or their PY-mutants (1-PY*, 2-PY*) were lysed and subjected to AQP2-immunoprecipitation (IP: α-AQP2). The AQP2 IP-fractions (upper panel) and lysates (lysate) were subjected to immunoblotting for NEDD4 or NEDD4L (IB: α-NEDD4/NEDD4L), NDFIP1/2/1-PY*/2-PY* (IB: α-myc), and AQP2 (IB: α-AQP2). Only upon co-expression with AQP2 and wild type NDFIP1/NDFIP2, NEDD4 or NEDD4L were detected in the immunoprecipitate. (B/C) *NEDD4/NEDD4L binding to NDFIP1-2 is needed for AQP2 ubiquitination and degradation*. HEK293 cells transiently expressed AQP2 and NEDD4 (B) or NEDD4L (C) with or without NDFIP1/NDFIP2 (1, 2) or their PY-mutants (1-PY*, 2-PY*). AQP2 was immunoprecipitated (IP: α-AQP2) and subjected to immunoblotting for ubiquitin (IB: α-Ub), revealing AQP2 coupled to one (AQP2-Ub_1_), two (AQP2-Ub_2_) or three (AQP2-Ub_3_) ubiquitin molecules. Semi-quantification of the ubiquitinated AQP2 signals is given (control is set to 100%). Co-transfection of AQP2 with NEDD4/NEDD4L and NDFIP1/NDFIP2 increased AQP2 ubiquitination, whereas with NDFIP1/2-PY mutants no increase was detected. Co-transfection of AQP2 with NEDD4 or NEDD4L alone does not increase the abundance of ubiquitinated AQP2. Immuno-precipitating IgGs ({IgG}) are indicated. Coomassie staining of the blots confirmed loading of protein equivalents. Molecular masses of proteins are indicated on the left (in kDa).

To investigate whether NDFIP1 or 2 could link NEDD4 or NEDD4L to AQP2, we transiently expressed AQP2 and NEDD4 or NEDD4L in HEK293 cells with or without myc-NDFIP1 or myc-NDFIP2. Besides, also the PY-mutants of the NDFIP proteins [14] were co-expressed, which lack all three PY motifs due to specific amino acid changes of the PY motifs (PXY changed into PAG [NDFIP1] or PXF [NDFIP2][14]). Following transfection and culturing of the HEK293 cells for 2 days, the cells were lysed and used for immunoblotting or subjected to AQP2 co-immunoprecipitation assays. Upon co-expression of NEDD4 or NEDD4L with wt-NDFIP1 or wt-NDFIP2, NEDD4 or NEDD4L clearly co-precipitated with AQP2 (Fig 3A, upper panels). Co-expression without NDFIP or the NDFIP PY-mutants prevented any NEDD4/NEDD4L co-precipitation or led to a strongly reduced interaction. Of importance, despite transfection of identical amounts of related constructs, the abundances of NEDD4/4L and NDFIP1/2 proteins were reduced in the lanes of the wt-NDFIP proteins as compared to their PY-mutated counterparts or, for the NEDD4/4L proteins, the control lanes (Fig 3A, lower panels). These data are in line with those of others that NEDD4 interaction affects the abundance of NDFIP1 [16] and, vice versa, that NDFIP1 and NDFIP2 interaction with NEDD4/4L reduces the abundance of the latter [14]. Although the E3 ligases and their adaptors mutually reduced their abundances, co-expression of NEDD4 or NEDD4L with NDFIP1 or NDFIP2, but not their non-interacting PY-mutated counterparts, decreased AQP2 abundance (Fig 3A). These results revealed that linking of NEDD4 or NEDD4L to AQP2 by NDFIP1 or NDFIP2 leads to degradation of AQP2.

To assess whether the reduced abundance of AQP2 upon interaction with NDFIP1/2 and NEDD4/4L coincided with increased ubiquitination of AQP2, AQP2 was co-expressed with NEDD4 or NEDD4L with and without NDFIP1, NDFIP2 or their PY-mutants and subsequently immunoprecipitated from HEK293 cell lysates. Again, on immunoblot wt-NDFIP1 was nearly invisible when co-trasnfected with NEDD4 or NEDD4L, despite transfection of equal amounts of construct, suggesting that co-expression of NDFIP and NEDD4/NEDD4L strongly reduces its expression, as discussed earlier. NEDD4 or NEDD4L alone did not lead to a remarkable change in ubiquitinated AQP2 (Fig 3B and 3C). However, when co-expressed with NDFIP1 or NDFIP2, but not their PY-mutants, a profound increase of ubiquitinated AQP2 was observed, despite a reduced total AQP2 abundance. These results revealed that NDFIP1/2-coupled NEDD4/NEDD4L complexes bind to and reduce AQP2 abundance by increasing its ubiquitination.

### NDFIP1 regulates AQP2 more strongly than NDFIP2 in mpkCCD cells

**Fig 4:**
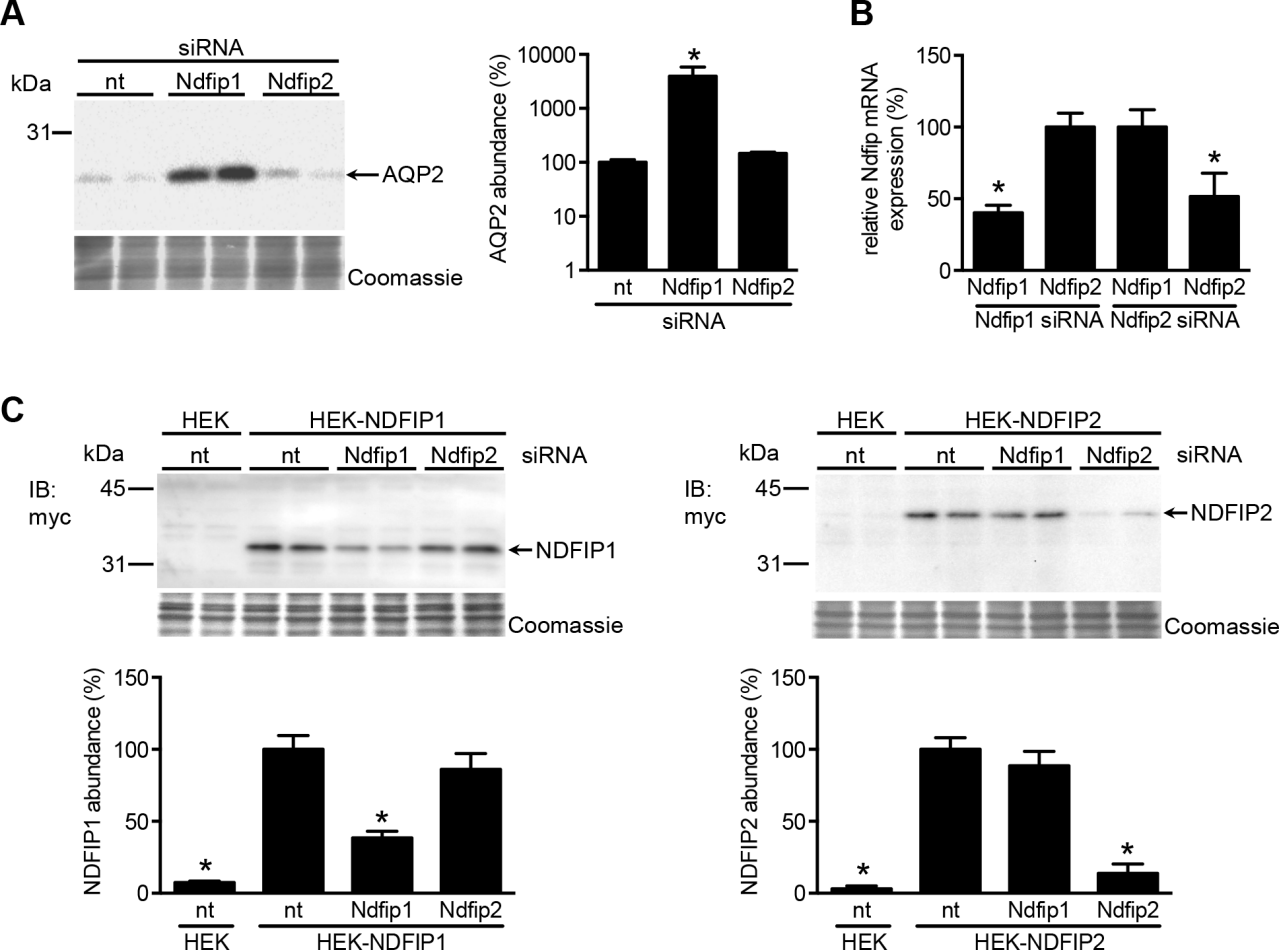
Downregulation of NDFIP1, but not NDFIP2, increases AQP2 abundance in mpkCCD cells. (A) MpkCCD cells were transfected with non-targeting siRNA (nt) siRNA against NDFIP1 and NDFIP2, grown for 4 days with dDAVP stimulation for the last 72h, and lysed. Subsequent immunoblotting for AQP2 and semi-quantification of the signals (n = 10 from 5 independent experiments) revealed that siRNAs against NDFIP1, but not NDFIP2, significantly increased the abundance of AQP2. (B/C) *NDFIP1 and NDFIP2 siRNAs specifically downregulate their mRNA and protein counterparts*. (B) RT-qPCR of mRNA isolated from transfected mpkCCD cells and normalized against GAPDH mRNA showed the NDFIP1 and NDFIP2 siRNAs conferred a significant and specific downregulation of their corresponding mRNAs (n = 3). mRNA amounts of cells transfected with siRNAs of the other NDFIP were used as controls and set to 100%. Asterisks indicate p<0.05 as compared to these controls. (C) To check for specific downregulation by the NDFIP siRNAs on protein level, HEK293 cells were left untransfected (HEK-nt) or transiently-transfected with expression constructs encoding NDFIP1 (HEK-NDFIP1) or NDFIP2 (HEK-NDFIP2). After 2 days of culture, the cells were transfected with non-targeting (nt), NDFIP1 or NDFIP2 siRNA, cultured for 2 days more, lysed, and subjected to NDFIP immunoblotting (IB: α-myc). The NDFIP signals were semi-quantified and compared to those of NDFIP-transfected cells treated with nt siRNAs, which was set to 100%. The NDFIP1 or NDFIP2 protein abundances were only and significantly reduced in HEK cells transfected with their respective siRNAs. Coomassie staining of the blots confirmed loading of protein equivalents. Molecular masses of proteins are indicated on the left (in kDa).

Transcriptome analyses revealed that both NDFIP1 and NDFIP2 are expressed in mpkCCD cells [17]. Therefore, to test a role of NDFIP1 or 2 in AQP2 regulation in a more physiological cell model, mpkCCD cells were incubated with dDAVP to induce AQP2 expression and treated with Ndfip1/2 siRNAs and non-targeting controls. Treatment with Ndfip1 siRNAs increased AQP2 abundance 40-fold compared to control siRNAs, whereas Ndfip2 siRNAs did not affect AQP2 abundance (Fig 4A). As we were not able to detect endogenous NDFIP1 or NDFIP2 in mpkCCD cells using different commercial antibodies (data not shown), we assessed their specific knockdown by RT-qPCR. Indeed, both siRNA pools showed similar levels of specific Ndfip1 or 2 mRNA downregulation (Fig 4B). To confirm effective knockdown of the NDFIP proteins by their specific siRNAs, we transiently-transfected HEK293 cells with myc-NDFIP1 or myc-NDFIP2 expression constructs in combination with non-targeting siRNA or either NDFIP siRNA. Subsequent immunoblot analysis revealed the specificity of the respective siRNAs, as only the NDFIP-expressing cells transfected with their corresponding siRNAs showed a significant reduction of NDFIP abundance (Fig 4C). Together, these data reveal that in the physiological collecting duct mpkCCD cell model, NDFIP1 reduces AQP2 abundance, in contrast to NDFIP2.

**Fig 5:**
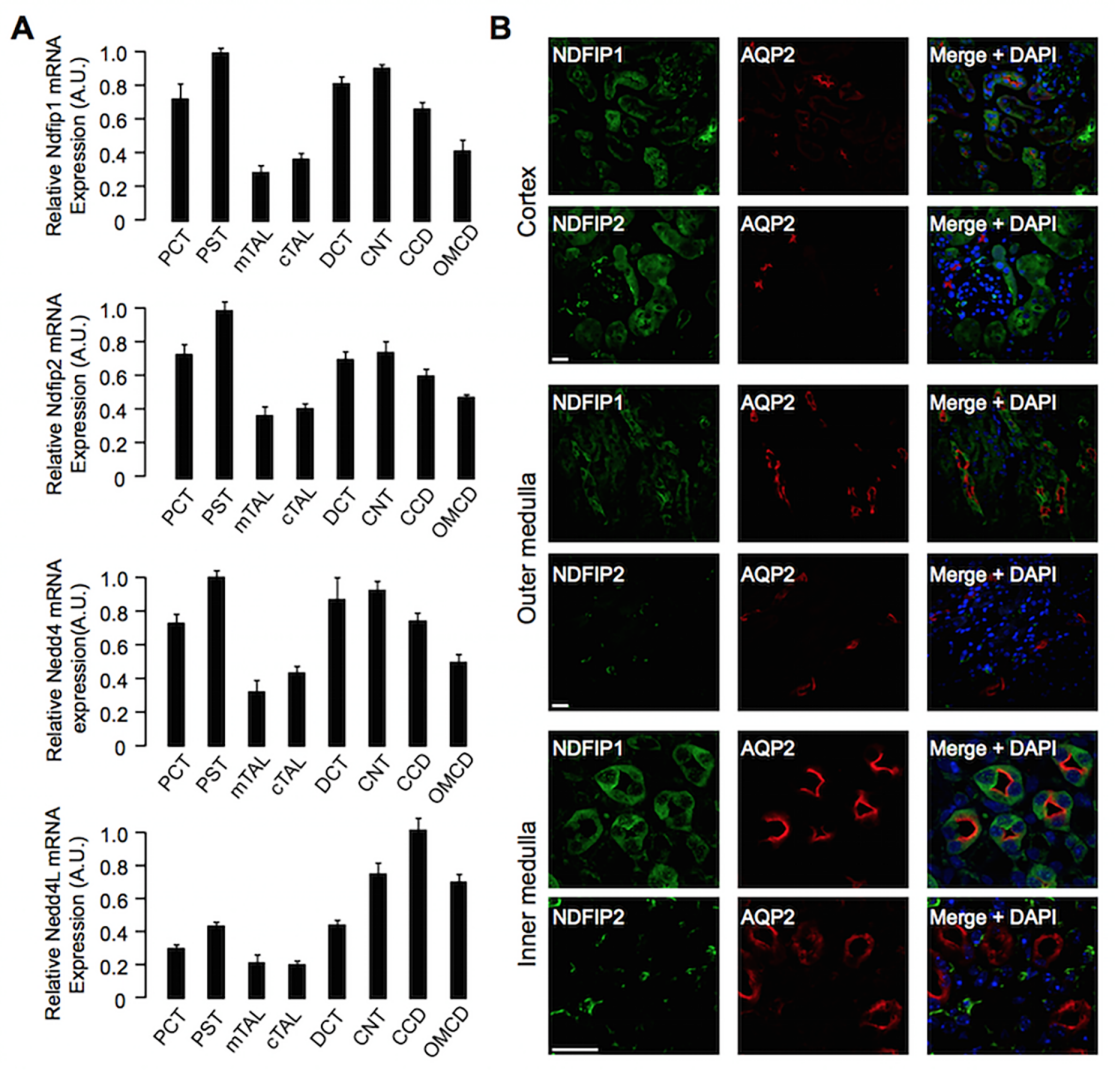
Expression of NDFIP1, NDFIP2, NEDD4 and NEDD4L in the mouse kidney. (A) *Ndfip1*, *Ndfip2*, *Nedd4 and Nedd4L mRNA expression along the nephron*. mRNA was isolated from different mouse nephron segments, and subjected to RT-qPCR to determine Ndfip1, Ndfip2, Nedd4 and Nedd4L mRNA levels. The determined mRNA levels were normalized to that of the housekeeping gene Rpl26 and presented as fraction of the segment with the highest level of expression, which was set to 1. Values are means ± SE from 6 mice. Segments indicated are proximal convoluted (S1) and straight (S3) tubule (PCT, PST), medullary (mTAL) and cortical (cTAL) thick ascending limb of Henle’s loop, distal convoluted tubule (DCT), connecting tubule (CNT), and the cortical (CCD) and outer medullary (OMCD) collecting duct. (B) *NDFIP1*, *but not NDFIP2*, *co-localizes with AQP2*. Cryo-sections of mouse kidneys were subjected to immunohistochemistry for NDFIP1 or NDFIP2 (green), AQP2 (red), and the nuclear dye DAPI (blue). Especially in the cortex and inner medulla, NDFIP1 colocalized with AQP2 in collecting duct principal cells. For NDFIP2, co-localization with AQP2 was never observed. Scale bars represent 25 μm.

To further establish a role for NDFIP1 and/or NDFIP2 in AQP2 regulation *in vivo*, we determined their mRNA and protein expression along the nephron. RT-qPCR analysis of mRNA isolated from different mouse nephron segments[18] and normalization against mRNA of the housekeeping Rpl26 gene revealed that Ndfip1 and Ndfip2 mRNAs were expressed in all segments tested, with highest expression of both in PCT, PST, DCT, CNT and CCD (Fig 5A). A similar mRNA distribution was found for Nedd4 and Nedd4L, except that expression of the latter was weak in PCT and PST (Fig 5A). To determine whether either NDFIP protein was co-expressed with AQP2 in principal cells, cryo-sections of kidneys of C57BL/6 mice were stained for NDFIP1 or NDFIP2, AQP2, and the nuclear marker DAPI. Especially in the cortex and inner medulla, NDFIP1 showed a dispersed staining in AQP2-positive collecting duct cells, whereas in the AQP2-negative intercalated cells of the collecting ducts, NDFIP1 localized in or near the apical membrane (Fig 5B). Moreover, NDFIP1 expression was also found in tubules negative for AQP2. In contrast, NDFIP2 showed a non-tubular staining in the inner medulla and outer medulla and did not co-localize with AQP2. In the cortex, NDFIP2 was also expressed in tubules, but did not co-localize with AQP2 (Fig 5B).

Co-localization studies with marker proteins of other tubular segments revealed a broad expression of NDFIP1 as it also co-stained with Aquaporin-1 (AQP1) in proximal tubules, but not descending loop of Henle, and Calbindin-28K in the DCT/CNT, whereas the expression of NDFIP2 was restricted to proximal tubules (co-staining with AQP1; S2 Fig). NDFIP1 and NDFIP2 were not detected in the thick ascending loop of Henle, indicated by the lack of co-staining with Tamm-Horsfall-Protein (THP).

## Discussion

### NDFIP1/2 are essential for NEDD4/NEDD4L to bind, ubiquitinate and degrade AQP2

Earlier, we showed that AQP2 ubiquitination is needed for its internalization from the plasma membrane and targeting for degradation [4]. Here, we provide compelling evidence that NEDD4/NEDD4L ubiquitin ligases ubiquitinate AQP2 and thereby induce its degradation. First, specific siRNA knockdown of NEDD4 and NEDD4L increased AQP2 abundance in polarized mpkCCD cells, a physiologically-relevant cell model of collecting duct principal cells (Fig 1). Also, in combination with NDFIP1/2, NEDD4 or NEDD4L were essential to increase AQP2 ubiquitination and degradation (Fig 3B and 3C). Consistent with our data, Lee at al. recently reported increased abundance of AQP2 in mpkCCD cells treated with NEDD4 siRNA [6].

However, while we could not detect direct binding of NEDD4 and NEDD4L with AQP2 (Fig 1C), our data indicate that either E3-ligase likely regulates AQP2 ubiquitination through NDFIP1 and/or NDFIP2. Indeed, we found that NDFIP1 and NDFIP2 directly interact with AQP2 and these NDFIP proteins are needed as adaptor proteins to link NEDD4/NEDD4L to AQP2 and to allow NEDD4/NEDD4L to ubiquitinate AQP2 (Figs 2 and 3). These NEDD4 family-interacting proteins (NDFIP) are integral membrane proteins (Fig 2B and 2C) that are potent activators of NEDD4 family members through multiple interactions of their PY elements with the NEDD4 WW domains. With this, they do not only sequester the E3s to endosomes, but also directly to their substrates, as shown for DMT1 and for several substrates of the yeast NDFIP ortholog Bsd2 [19]. The essential involvement of both NEDD4/NEDD4L and NDFIP1/2 in the ubiquitination and consequent degradation of AQP2 is further supported by our data that these effects on AQP2 were absent with NDFIP1/2 proteins lacking the PY elements (Fig 3) and that treatment of mpkCCD cells with NDFIP1 siRNA increased AQP2 abundance (Fig 4).

At present, however, it is unclear whether NDFIP1 and/or NDFIP2 is of relevance in the *in vivo* NEDD4/4L regulation of AQP2 ubiquitination and degradation. While our HEK293 co-immunoprecipitation studies, showing more AQP2 pulldown with NDFIP2 (Figs 2D and 3A), could suggest that NDFIP2 interacts better with AQP2, the reduced abundance of pulled down NDFIP1 may be a reflection of its reduced total abundance (lysate IB a-myc lanes). Besides, there is no reason to assume that a physiological relevant interacting protein should bind stronger. Our mpkCCD cells siRNA experiments clearly reveal that NDFIP1 was expressed endogenously and that its specific knockdown resulted in increased AQP2 abundance (Fig 4), underscoring the relevance of NDFIP1 in AQP2 regulation in a generally-accepted physiological principal cell model. Despite the earlier detection of NDFIP1 and NDFIP2 mRNA in mpkCCD cells [17] and the proof that the used NDFIP2 siRNA effectively and specifically knocked down NDFIP2 mRNA, the absence of an effect of NDFIP2 siRNA knockdown on AQP2 stability could be explained by an absence or low level of expression of NDFIP2 in mpkCCD cells. Our segment mRNA profiling and immunohistochemistry are suggestive of a role of NDFIP1, rather than of NDFIP2, in regulating AQP2 in principal cells. Immunohistochemistry revealed clear NDFIP1, but not NDFIP2, staining in principal cells of the entire collecting duct (Fig 5B). The observed ‘cytosolic’ localization of NDFIP1, rather than plasma membrane or nuclear localization, is consistent with its reported main localization in the trans Golgi Network [16, 20]. Moreover and consistent with our mRNA profiling, NDFIP1 staining was observed in proximal tubules and absent in mTAL and cTAL. Although protein abundance does not necessarily reflect mRNA expression, NDFIP1 staining was not observed in the DCT and CNT, which have relatively high NDFIP1 mRNA expression levels. Our NDFIP2 immunostaining was only partially consistent with our mRNA profiling, as our antibodies only stained the proximal tubules. As these segments show the relative highest mRNA expression of NDFIP2 (Fig 5A), our NDFIP2 antibodies may only stain the segments with the highest expression. The presence of NDIP2 in other segments is supported by data of Konstas et al., whose antibodies detected abundant NDFIP2 in DCT, CNT, CCD, OMCD and IMCD [13]. Furthermore, the expression of NDFIP2 in these segments, which also express its interacting partner ENaC, is consistent with the identification that a single nucleotide polymorphism in NDFIP2 correlates with hypertension [21]. The staining of Konstas et al. is in line with our mRNA profiling, except that they did not detect NDFIP2 in proximal tubules, which show the highest NDFIP2 expression in our mRNA profiling. While omission of our primary antibodies did not reveal any immunohistochemical staining, our NDFIP antibodies were unable to detect their respective NDFIP proteins by immunoblotting and, therefore, the segment-specific renal localization of NDFIP1/2 awaits development of improved NDFIP1/2 antibodies.

Based on our mRNA profiling and data of others, NEDD4 and NEDD4L may be involved in NDFIP-mediated ubiquitination of AQP2 *in vivo*, as both are expressed in renal principal cells [22, 23]. NEDD4L has been demonstrated to be the critical regulator of the principal cell epithelial sodium channel ENaC and blood pressure [24, 25]. Moreover, NEDD4, but not NEDD4L, was found in AQP2-containing vesicles isolated from rat inner medulla’s, and, consistent with a role in AQP2 ubiquitination and degradation, its expression was increased with a 6 hrs withdrawal of dDAVP [6].

### Possible roles of NDFIP1/2 in targeting AQP2 for degradation

Earlier, we have shown that K63-linked ‘mono’-ubiquitination of AQP2 is needed for an enhanced internalization of AQP2 from the plasma membrane and its targeting for lysosomal degradation [26]. Considering the role of NEDD4/4L in the regulation of the plasma membrane expression of the epithelial sodium channel ENaC, which is also expressed in principal cells [27], it is tempting to assume that NEDD4/4L and NDFIP1/2 ubiquitinate AQP2 and thereby directly regulate the plasma membrane abundance of AQP2. This, however, may not be necessarily the case. It has been shown that in the endocytic sorting pathway, monoubiquitinated cargo proteins are clustered by the endosomal sorting complexes required for transport (ESCRT) and delivered to vesicles that invaginate into the lumen of multivesicular bodies (MVBs), eventually resulting in lysosomal degradation [28, 29]. Non- or deubiquitinated proteins might exit this pathway in an early stage or will remain in the MVB limiting membrane, from where they may recycle. Moreover, it becomes more evident that membrane proteins can targeted for degradation following their ubiquitination at intracellular organelles. In non-activated dendritic cells, for example, peptide-loaded Major Histocompatibility Complex (MHC)II is ubiquitinated at endosomes and consequently sorted by the ESCRT machinery to intraluminal vesicles of multivesicular bodies, ultimately leading to its lysosomal degradation [30]. More specifically and consistent with the ‘intracellular’ localization of at least NDFIP1 in principal cells (Fig 5B), NDFIP1 and NDFIP2 have mainly been localized to the Golgi apparatus, late endosomes and MVBs, and have been shown to regulate the plasma membrane expression of the divalent ion metal transporter DMT1 by ubiquitinating DMT1 at the Golgi followed by lysosomal targeting [16, 20, 31–33]. As such, it remains to be established whether ubiquitination of AQP2 and its targeted degradation by NDFIP1/2-NEDD4/4L interaction occurs at the plasma membrane or an intracellular organelle. Moreover, as NEDD4-mediated ubiquitination has been shown to also affect transcription of genes [34], we cannot exclude that NDFIP1/2-NEDD4/4L may also have affected (endogenous) AQP2 gene transcription in mpkCCD. This is less likely for our transfected HEK293 cell experiments, as there AQP2 transcription is regulated by a standard cytomegalovirus promoter.

In conclusion, we demonstrated here that the E3-ligases NEDD4 and NEDD4L can regulate AQP2 ubiquitination and degradation, but only when they are recruited to AQP2 via adaptor proteins NDFIP1 or NDFIP2. As NDFIP1 and NDFIP2 bind many NEDD4 family of HECT domain E3 ligases, which are implicated in a wide variety of cellular processes, such as differentiation (ITCH) and signalling (SMURF1/2 and ITCH) [32], NDFIP1/2 interacting with AQP2 may attenuate AQP2 expression and abundance at many levels, depending on the E3 ligase interacting with NDFIP1/2. Moreover, our data may open a new view on the differential regulation of water and sodium reabsorption in collecting ducts, since our data suggests that, besides ENaC, also AQP2 may be regulated by NEDD4L in principal cells. The binding of NDFIP1/2 to NEDD4L, which can be modulated by phosphorylation upon hormonal stimulation, could determine the availability of NEDD4L to either regulate sodium transport via ENaC or water transport via AQP2.

## Methods

### Membrane yeast two- hybrid (MYTH) assay

Full-length human AQP2 and kidney cDNA library were used in membrane yeast two hybrid assays as described [35]. The assay and construct generation was performed as described [35]. Template cDNA of human AQP2 was amplified by PCR [36]. Subsequently the cDNA was digested with *Xba*l and *BspH*1, and ligated in the *Xba*l and *Nco*l sites of pTMBV, resulting in a C-terminal Cub-tagged AQP2 construct. The triple *S*. *cerevisiae* reporter strain (HIS3/ADE2/LacZ) NMY51 (Dualsystems Biotech, Schlieren, Switzerland) was transformed with pTMBV-hAQP2-Cub. Proper localization in the yeast’s plasma membrane was confirmed by expression of pTMBV-hAQP2-Cub with YFP cloned in-frame to the N-terminal end of hAQP2. For this, the YFPcDNA was amplified from the pCYT-L3 plasmid [37] by PCR (Fwd5' ctaagaggtggtatgcacagatcagcttgcggccgcagtaaaggagaagaacttttcactg 3', Rev 5' gatcaaacacctcttgttgcctggccgttaacgctttcatgcggccgcctttgtatagttc 3'). pTMBV-hAQP2-Cub was digested with *Not*I. Both the PCR product and the digested bait plasmid were co-transformed into the NMY51 strain and allowed to homologously recombine to generate the hAQP2-Cub-YFP construct. Plasma membrane expression of AQP2-YFP was confirmed by fluorescence microscopy using a Leica DMI 6000 B Inverted Confocal Microscope with YFP and differential interference contrast (DIC) filter sets (S1A Fig). For image acquisition and processing, the Volocity software package was used (PerkinElmer).

The absence of self-activation was assessed by transformation of the bait strains with interacting (NubI, positive control) or non-interacting (NubG, negative control) control proteins, being the membrane proteins oligosaccharyltransferase 1 (OST1) and uracil permease-4 (FUR4), which localize to the endoplasmatic reticulum and plasma membrane, respectively. A bait must grow on selective medium in the presence of the positive control, but not in the presence of the negative control, to be suitable for MYTH. The pTMBV-hAQP2-Cub fulfils these criteria (S1B Fig). For screening, a NubG-tagged human kidney cDNA library (Dualsystems Biotech, Schlieren, Switzerland) was transformed in the bait yeast strain and cells were grown on transformation selective media plates (SD-WL, lacking tryptophan [W] and leucine [L]) and interaction selective media plates (SD-WLAH, lacking tryptophan, leucine, adenine and histidine) containing X-Gal. Of robustly growing blue colonies, DNA was isolated and sequenced. From these clones, genuine interaction was confirmed by back transformation with the original pTMBV-hAQP2-Cub construct, or, as a negative control, the sequence of the transmembrane domain of CD4 coupled to Cub (referred to as the ‘artificial bait’).

### Cell culture and transfection

HEK293 cells were grown in DMEM (Lonza, Verviers, Belgium) supplemented with 5% FCS, 2 mM glutamine and non-essential amino acids (PAA Laboratories, Linz, Austria). For transfection, cells were seeded at 1.5*10^6^ cells/well in 6-well culture plates (Corning Costar, Cambridge, USA) and allowed to attach for 4 hours prior to transfection. Transient transfection was performed with 2 μg DNA for 1.5*10^6^ cells and 12 μl PEI (Polysciences, Warrington, PA, USA) as transfection reagent. Before harvesting after 48 hours, the transfected cells were treated with 10 nM TPA (Sigma, St. Louis, MO, USA) for 15 min to induce AQP2 ubiquitination as described [4].

MpkCCD cells (clone 11) were cultured as described [38]. Cells were seeded with a density of 1.5*10^5^ cells/cm^2^ on 24-well (0.33 cm^2^) semi-permeable filters (Costar Corning Transwell®, 0.4 μm pore size) and grown for 4 days. For the last 72 hours, 1 nM dDAVP (Sigma, St. Louis, MO, USA) was added to the medium at the basolateral side to stimulate expression of endogenous AQP2.

For (transient) siRNA knockdown in mpkCCD cells, siGENOME SMARTpool (Thermo Fisher Scientific, Lafayette, CO, USA) siRNAs were obtained against mouse NDFIP1 (NM_022996), NDFIP2 (NM_029561), NEDD4 (NM_010890), NEDD4L (NM_001114386) and a scrambled non-targeting siRNA as a control (for siRNA sequences, see S2 Table). 0.5*10^5^ mpkCCD cells were seeded per 24 well filters and transfected with 20 pmol siRNA, combined with 1 μl Metafectene Pro (Biontex, Martinsried, Germany) at day 1. After 4 days, cells were harvested and prepared for immunoblotting.

Human AQP2 was expressed from a pCB6-d*Bam*HI-AQP2 construct as described [39]. psDeasy-human-NEDD4 and -mouse-NEDD4L (gift from Prof. Staub, Switzerland) are described [40]. For expression in mammalian cells, hNEDD4 and mNEDD4L cDNAs were cut from the psDeasy constructs with *Xho*I*/Msc*I and *EcoR*I*/Sac*II, respectively, and cloned into pLV-CMV digested with *Sma*I, *Xho*I*/Nhe*I and *EcoR*I*/Sac*II, respectively. PcDNA3.1 expression constructs encoding wildtype (wt) mouse NDFIP1, NDFIP2 or their PY-motif mutants are as described [14] and were provided by Prof. Pelham (UK).

### Reverse transcription-quantitative PCR (RT-qPCR)

Total RNA from siRNA-transfected mpkCCD cells was isolated using TRIzol® (Gibco Life Technologies, Rockville, MD, USA) and 1.5 μg of RNA was reverse transcribed with an Moloney murine leukemia virus (M-MLV) reverse transcriptase kit (Promega, Madison, WI, USA), both according to the suppliers’ protocol. Following a 10-fold dilution, mRNA expression levels of NDFIP1 and NDFIP2 were determined by RT-qPCR using SYBR-green (Applied Biosystems, Foster City, CA, USA) and were normalized to the housekeeping gene GAPDH. Primer sequences were as listed in S3 Table.

### Immunoprecipitation and co-immunoprecipitation

For AQP2 immunoprecipitation, HEK293 cells of one 6-well, transiently-transfected for 48 hours, were lysed in 500 μl lysis buffer (125 mM NaCl, 25 mM HEPES, 1% Triton, pH 7.5), supplemented with the following proteinase, phosphatase and deubiquitination inhibitors: 20 mM N-ethylmaleimide, 10 mM NaF, 1 mM PMSF, 0.5 mM Na_3_VO_4_ (all Sigma, St. Louis, MO, USA), and 10 μg/ml aprotinin, 10 μg/ml leupeptin, 2 μg/ml pepstatin (all MP Biomedicals Illkirch, France). For co-immunoprecipitation experiments, cells were lysed in 500 μl co-IP lysis buffer (100 mM NaCl, 25 mM Tris-HCl, 1 mM EDTA, 1 mM MgCl_2_, 1 mM CaCl_2_, 0.3% NP40, pH 7.5) supplemented with the inhibitors above.

Before immunoprecipitation, cell debris was removed by centrifugation at 16100 g for 10 min. 25 μl Protein A/G-Agarose beads (Santa Cruz Biotechnology, Santa Cruz, CA, USA) were washed three times in the appropriate lysis buffer followed by incubation with 10 μl rabbit-anti-AQP2 antibody directed against the pre-C tail [41] for 4 hours at room temperature and washed again three times with inhibitor-supplemented lysis buffer. Lysate of one 6-well was added to the beads and incubated overnight at 4°C, followed by three washing steps with lysis buffer. Immunoprecipitated protein was eluted from beads in 60 μl inhibitor-supplemented laemmli buffer with 100 mM DTT (MP Biomedicals, Illkirch, France) by incubation at 37° C for 30 min after which samples were subjected to immunoblot analyses.

### Analysis of mRNA expression along the mouse nephron

The different nephron segments were dissected from collagenase-treated kidneys of male C57Bl6/J mice as previously described [42]. Total RNAs were extracted using RNeasy micro kit (Qiagen, Hilden, Germany) from pools of ~50 nephron segments of which the total length was determined by image analysis (Visilog, Noesis, France). RNAs were reverse transcribed using a first strand cDNA synthesis kit for RT-PCR (Roche Diagnostics), according to the manufacturers' protocols. Real-time PCR was performed using a cDNA quantity corresponding to 0.1 mm of nephron segments with LightCycler 480 SYBR Green I Master qPCR kit (Roche Diagnostics) according to the manufacturer's protocol. Specific primers (S3 Table) were designed using ProbeDesign (Roche Diagnostics). In each experiment, a standardization curve was made using serial dilutions of a standard cDNA stock solution made from whole kidney RNA. The amount of PCR product was calculated as percent of the standard DNA and gene expression was normalized as a function of that of the housekeeping gene Rpl26. Results are means ± SE from 6 mice.

### Immunoblotting and immunohistochemistry

Immunoblotting and immunohistochemistry was done as described [4, 43]. For immunoblotting, rabbit anti-AQP2 [44], mouse-anti-myc and anti-ubiquitin (Sigma, St. Louis, MO, USA), rabbit anti-NEDD4 [22], rabbit anti-NEDD4L [45] and rabbit-anti-NDFIP1 (Abcam, Cambridge, UK)) antibodies were used. For immunohistochemistry, kidneys from 12-weeks old C157BL/6 mice were used and stained with rabbit anti-NDFIP1 or NDFIP2 (Abcam, Cambridge, UK) together with guinea-pig anti-AQP2 (1:300) [44], sheep anti-Tamm-Horsfall Protein (Biotrend, Cologne, Germany), mouse-anti-Calbindin 28K (Sigma) or mouse anti-AQP1 [46] antibodies. Immunoblots were densitrometrically analysed using Bio-Rad quantification equipment (Bio-Rad 690c Densitometer; Chemidoc XRS) and software (QuantityOne; Bio-Rad). Signals were normalized to loading control (Coomassie staining).

### Study approval

All animal studies were approved by the Animal Ethical Committee of the Radboud University Medical Center.

## Supporting information

S1 Fig Localization and self-dependency test of bait pTMBV-hAQP2 in yeast cells. (A) The membrane localization of the bait used in the MYTH-assay (pTMBV-hAQP2) was confirmed by inclusion of YFP in the AQP2 construct and fluorescence microscopy. Arrows indicated membrane localization in pTMBV-YFP-hAQP2 transformed yeast cells. No fluorescence was detected in yeast cells transformed with empty pTMBV. (B) The level of self-activation was assessed by transformation of the bait strains with an interacting (NubI) or non-interacting (NubG) control, namely the endoplasmatic reticulum membrane protein oligosaccharyltransferase (OST1) and the uracil permease FUR4, localized to the plasma membrane. No self-activation was detected, indicated by the lack of colony growth with OST1-NubG or FUR4-NubG on interaction selective media.
(TIFF)

S2 Fig Expression of NDFIP1 and NDFIP2 in mouse kidney. Cryosections of C57/BL6 mouse kidney were stained for NDFIP1 and NDFIP2 and co-stained with marker proteins for different kidney sections. (A) NDFIP1 was detected in proximal tubules (co-staining with AQP1), and the distal convoluted tubule (co-staining with Calbindin 28K). (B) NDFIP2 was only detected in the proximal tubules where it showed co-staining with AQP1. Scale bars represent 25 μm.
(PDF)

S1 Table Identified proteins in AQP2 MYTH. (PDF)

S2 Table Used SMARTpools siRNA sequences. (PDF)

S3 Table Primers used for RT-qPCR analyses. (PDF)
