# Supplementary figures and images for: NDFIP allows NEDD4/NEDD4L-induced AQP2 ubiquitination and degradation
Source: PLoS One. 2017 Sep 20;12(9):e0183774. doi: 10.1371/journal.pone.0183774 (PMC5606929; doi:10.1371/journal.pone.0183774)

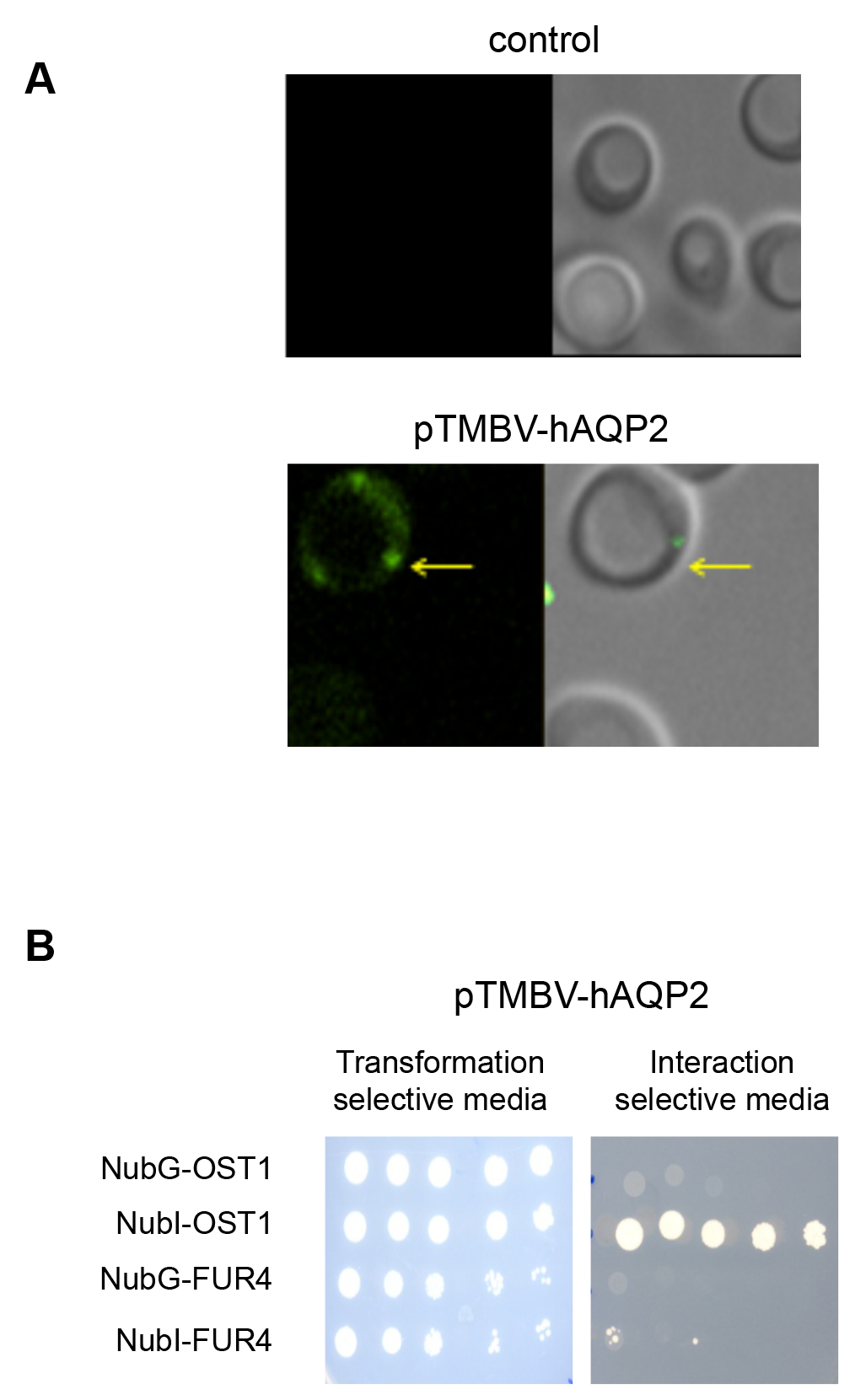

Supplement: S1 Fig — (A) The membrane localization of the bait used in the MYTH-assay (pTMBV-hAQP2) was confirmed by inclusion of YFP in the AQP2 construct and fluorescence microscopy. Arrows indicated membrane localization in pTMBV-YFP-hAQP2 transformed yeast cells. No fluorescence was detected in yeast cells transformed with empty pTMBV. (B) The level of self-activation was assessed by transformation of the bait strains with an interacting (NubI) or non-interacting (NubG) control, namely the endoplasmatic reticulum membrane protein oligosaccharyltransferase (OST1) and the uracil permease FUR4, localized to the plasma membrane. No self-activation was detected, indicated by the lack of colony growth with OST1-NubG or FUR4-NubG on interaction selective media. (TIFF) [file pone.0183774.s001.tiff]
