# Supplementary material for: NDFIP allows NEDD4/NEDD4L-induced AQP2 ubiquitination and degradation
Source: PLoS One. 2017 Sep 20;12(9):e0183774. doi: 10.1371/journal.pone.0183774 (PMC5606929; doi:10.1371/journal.pone.0183774)
Supplement: S2 Fig — Cryosections of C57/BL6 mouse kidney were stained for NDFIP1 and NDFIP2 and co-stained with marker proteins for different kidney sections. (A) NDFIP1 was detected in proximal tubules (co-staining with AQP1), and the distal convoluted tubule (co-staining with Calbindin 28K). (B) NDFIP2 was only detected in the proximal tubules where it showed co-staining with AQP1. Scale bars represent 25 μm. (PDF) [file pone.0183774.s002.pdf]

Figure S2

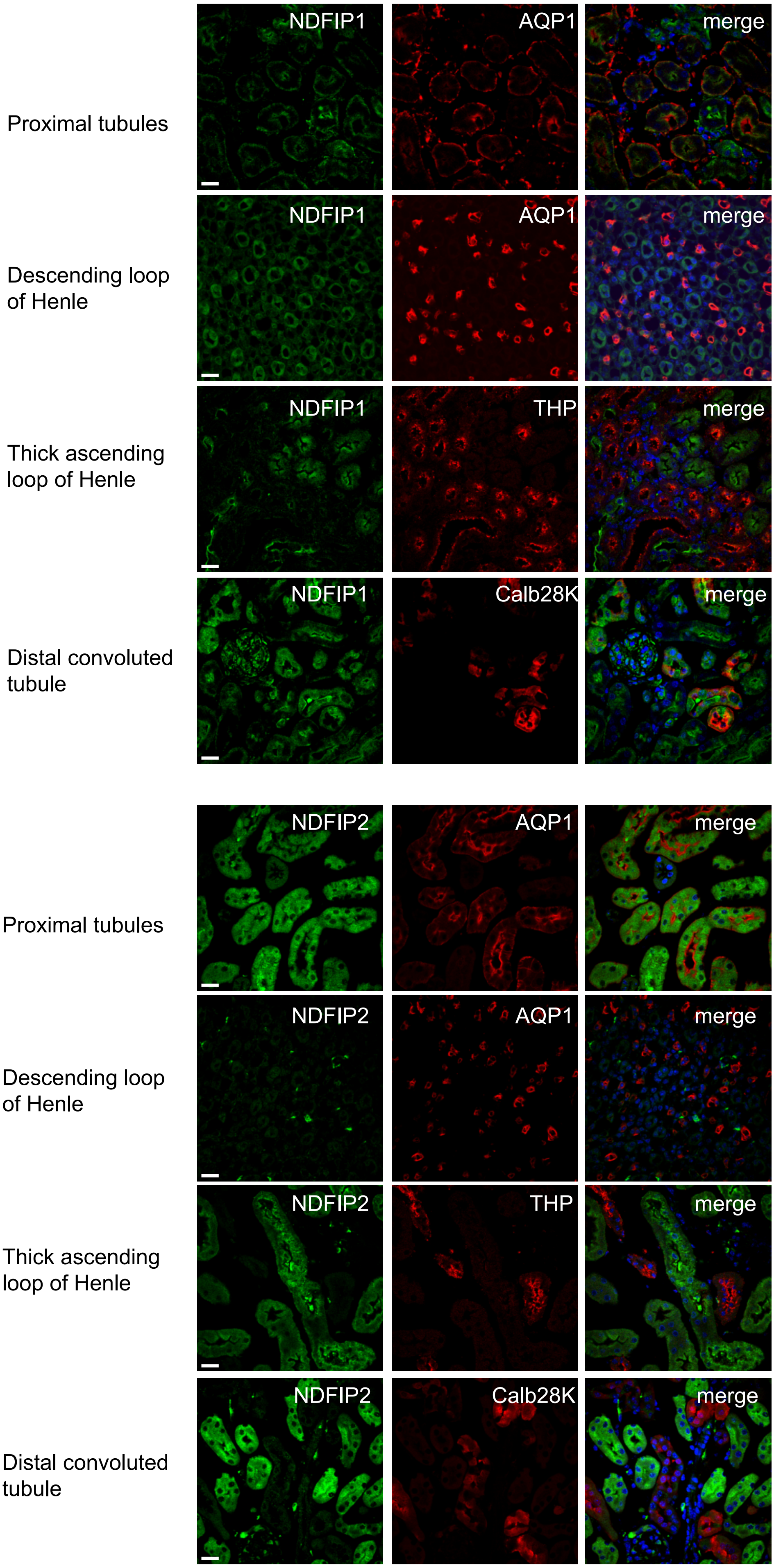
Proximal tubules
NDFIP1
AQP1
merge
Descending loop of Henle
NDFIP1
AQP1
merge
Thick ascending loop of Henle
NDFIP1
THP
merge
Distal convoluted tubule
NDFIP1
Calb28K
merge
Proximal tubules
NDFIP2
AQP1
merge
Descending loop of Henle
NDFIP2
AQP1
merge
Thick ascending loop of Henle
NDFIP2
THP
merge
Distal convoluted tubule
NDFIP2
Calb28K
merge
