# Supplementary material for: NDFIP allows NEDD4/NEDD4L-induced AQP2 ubiquitination and degradation
Source: PLoS One. 2017 Sep 20;12(9):e0183774. doi: 10.1371/journal.pone.0183774 (PMC5606929; doi:10.1371/journal.pone.0183774)
Supplement: S1 Table — (PDF) [file pone.0183774.s003.pdf]

# Table S1. Identified proteins in AQP2 MYTH

| Bait | Name | Alternative names |
|---|---|---|
| pTMBD-hAQP2wt | Homo sapiens syntaxin 8 (STX8), transcript variant 1, mRNA | CARB |
| pTMBD-hAQP2wt | Homo sapiens ubiquitin C (UBC), mRNA | HMG20 |
| pTMBD-hAQP2wt | Homo sapiens immediate early response 3 (IER3), mRNA | DIF2; IEX1; PRG1; DIF-2; GLY96; IEX-1; IEX-1L |
| pTMBD-hAQP2wt | Homo sapiens aquaporin 2 (collecting duct) (AQP2), mRNA | |
| pTMBD-hAQP2wt | Homo sapiens Nedd4 family interacting protein 2 (NDFIP2), transcript variant 1, mRNA | N4WBP5A; FLJ25842; KIAA1165 |
| pTMBD-hAQP2wt | Homo sapiens B-cell receptor-associated protein 31 (BCAP31), transcript variant 4, non-coding RNA | CDM; BAP31; 6C6-AG; DXS1357E |
| pTMBD-hAQP2wt | Homo sapiens chromosome 20 open reading frame 30 (C20orf30), transcript variant 4, mRNA | |
| pTMBD-hAQP2wt | Homo sapiens signal peptidase complex subunit 1 homolog (S. cerevisiae) (SPCS1), mRNA | SPC1; SPC12; HSPC033; YJR010C-A |
| pTMBD-hAQP2-K270R | Homo sapiens proteolipid protein 2 (colonic epithelium-enriched) (PLP2), mRNA | A4; A4LSB; MGC126187 |
| pTMBD-hAQP2-K270R | Homo sapiens ubiquitin-conjugating enzyme E2, J1, U (UBE2J1), mRNA | UBC6; Ubc6p; CGI-76; NCUBE1; HSPC153; HSPC205; NCUBE-1; HSU93243; MGC12555 |
| pTMBD-hAQP2-K270R | Homo sapiens coiled-coil domain containing 167 (CCDC167), mRNA | |
| pTMBD-hAQP2-K270R | Homo sapiens chromosome 14 open reading frame 1 (C14orf1), mRNA | |
| pTMBD-hAQP2-K270R | Homo sapiens signal sequence receptor, gamma (translocon-associated protein gamma) (SSR3), mRNA | TRAPG |
| pTMBD-hAQP2-K270R | Homo sapiens collagen, type IV, alpha 3 (Goodpasture antigen) binding protein (COL4A3BP), transcript variant 1, mRNA | CERT; GPBP; CERTL; STARD11; FLJ20597 |
| pTMBD-hAQP2-S261D | Homo sapiens claudin 7 (CLDN7), mRNA | CLDN-7; CEPTRL2; CPETRL2; Hs.84359; claudin-1 |
| pTMBD-hAQP2-S261D | Homo sapiens actin, beta (ACTB), mRNA | PS1TP5BP1 |
| pTMBD-hAQP2-S261D | Homo sapiens chromosome 4 open reading frame 3 (C4orf3), transcript variant 2, mRNA | |
| pTMBD-hAQP2-S261D | Homo sapiens signal peptidase complex subunit 1 homolog (S. cerevisiae) (SPCS1), mRNA | SPC1; SPC12; HSPC033; YJR010C-A |
| pTMBD-hAQP2-S256D-S264D-T269E | Homo sapiens eukaryotic translation elongation factor 1 alpha 1 (EEF1A1), mRNA | CCS3; EF1A; PTI1; CCS-3; EE1A1; EEF-1; EEF1A; EF-Tu; LENG7; eEF1A-1; FLJ25721; GRAF-1EF |
| pTMBD-hAQP2-S256D-S264D-T269E | Homo sapiens solute carrier family 5 (sodium/glucose cotransporter), member 2 (SLC5A2), mRNA | SGLT2 |
| pTMBD-hAQP2-S256D-S264D-T269E | Homo sapiens sorting nexin 5 (SNX5), transcript variant 1, mRNA | FLJ10931 |
| pBT3-hAQP2-S256D-S264D-T269E | Homo sapiens elongation factor, RNA polymerase II, 2 (ELL2), mRNA | |
| pBT3-hAQP2-S256D-S264D-T269E | Homo sapiens VAMP (vesicle-associated membrane protein)-associated protein B and C (VAPB), transcript variant 1, mRNA | ALS8; VAP-B; VAMP-B |
| *pBT3-hAQP2-S256D-S264D-T269E* | FAM48A (family with sequence similarity 48, member A) | C13; FP757; P38IP; SPT20; C13orf19; bA421P11.4 |
| pBT3-hAQP2-S256D-S264D-T269E | TM9SF2 (transmembrane 9 superfamily member 2) | P76 |
| pBT3-hAQP2-S256D-S264D-T269E | GPAA1 glycosylphosphatidylinositol anchor attachment protein 1 homolog | GAA1; hGAA1 |
