# Supplementary material for: NDFIP allows NEDD4/NEDD4L-induced AQP2 ubiquitination and degradation
Source: PLoS One. 2017 Sep 20;12(9):e0183774. doi: 10.1371/journal.pone.0183774 (PMC5606929; doi:10.1371/journal.pone.0183774)
Supplement: S2 Table — (PDF) [file pone.0183774.s004.pdf]

Table S2. Sequences of siRNA SMARTpools (Thermo Fisher Scientific, Lafayette, CO, USA)

Gene Symbol	Gene Accession	siRNA sequences (pool of 4)
Nedd4	NM_010890	GGAAGGACCUACUACGUAA
		GGGCGAGUCUUCUUCAUAA
		AGACUGACAUUCCAAACAA
		UAAUUACACCCUACAGAUA
Nedd4L	NM_001114386	CGACCCAGCUUGAUGGAUG
		AGUCAUAAAUCUCGAGUCA
		CUUCGGUCCUGCAGUGUUA
		CGGAGGAUCAUGUCUGUAA
Ndfip1	NM_022996	GAACCAAGACUGAAGCUAC
		GGAUUUAUCAAUUACGCAA
		GUUCGGAAGAUGCCAGAAA
		GCUGAGGAUAGGAAACGAU
Ndfip2	NM_029561	CGGGAUGACUUCAGUGAUG
		UACCAUCGCUGGAAGAUAC
		CUAUUUCAAUGGACAGUAU
		GGUACUUCUUCCUGCUAUA
