# Supplementary material for: NDFIP allows NEDD4/NEDD4L-induced AQP2 ubiquitination and degradation
Source: PLoS One. 2017 Sep 20;12(9):e0183774. doi: 10.1371/journal.pone.0183774 (PMC5606929; doi:10.1371/journal.pone.0183774)
Supplement: S3 Table — (PDF) [file pone.0183774.s005.pdf]

Table S3. Primer sequences

Protein	forward primer 5'...3'	reverse primer 5'…3'
NDFIP1	GCGGGTACCAGCAGTTGCAGAAT	GCAGTGATGCTGCTGTATGGTGGA
NDFIP2	GGTGCTATCTGTGGGTTTGGCCTTT	GAAAAGGAGCAGGCCAAGTACGAGA
GAPDH	TAACATCAAATGGGGTGAGG	GGTTCACACCCATCACAAAC
